# Development of 3D printed patient-specific skull implants based on 3d surface scans

**DOI:** 10.1186/s41205-023-00183-x

**Published:** 2023-06-30

**Authors:** Fabian Kropla, Dirk Winkler, Dirk Lindner, Patrick Knorr, Sebastian Scholz, Ronny Grunert

**Affiliations:** 1 Department of Neurosurgery, University of Leipzig, 04103 Leipzig, SN Germany; 2 Department of Neurosurgery, University of Leipzig Medical Center, Liebigstr. 20, 04103 Leipzig, Germany; 3 Department of Neurosurgery, University Hospital Leipzig, Liebigstr. 20, 04103 Leipzig, Germany; 4 Department for Automotive and Mechanical Engineering, University of Applied Sciences Zwickau, 08056 Zwickau, SN Germany; 5 Fraunhofer Institute for Machine Tools and Forming Technology, 02763 Zittau, SN Germany

**Keywords:** Cranioplasty, Neurosurgery, 3D scan, Patient-specific implant creation, 3D printing, Additive manufacturing

## Abstract

Sometimes cranioplasty is necessary to reconstruct skull bone defects after a neurosurgical operation. If an autologous bone is unavailable, alloplastic materials are used. The standard technical approach for the fabrication of cranial implants is based on 3D imaging by computed tomography using the defect and the contralateral site. A new approach uses 3D surface scans, which accurately replicate the curvature of the removed bone flap. For this purpose, the removed bone flap is scanned intraoperatively and digitized accordingly. When using a design procedure developed for this purpose creating a patient-specific implant for each bone flap shape in short time is possible. The designed skull implants have complex free-form surfaces analogous to the curvature of the skull, which is why additive manufacturing is the ideal manufacturing technology here. In this study, we will describe the intraoperative procedure for the acquisition of scanned data and its further processing up to the creation of the implant.

## Introduction

### Medical overview

Cranioplasty is routinely used to cover a cranial bone defect after space-occupying cerebral ischemia, traumatic brain injury, intracerebral or subarachnoid haemorrhage, and infection in neurosurgery [1]. For this purpose, decompression represents the last step of life-saving intervention [2]. After the brain swelling has subsided, the removed bone flap can be reimplanted in most cases [3]. This operation requires a second surgical procedure to reconstruct the skull, which occurs at a particular time interval from the first craniotomy [4].

When it is not possible to use the human bone flap, artificial materials, so-called alloplastic materials, are used. The success of this operation depends on several factors, such as the size and position of the cranioplasty, the patient's condition, the material used, and the implantation time [5]. In the worst case, infection, prolonged hospitalization, implant loss, and even mortality can follow [6].

For example, in a study of 754 cranioplasty patients, the rate for an alloplastic implant was 29.4% (*n* = 222) [7], with a complication rate in a quarter of the patients. In this paper, the time between removal of the bone flap and the final implant placement has shown to have had a significant influence. The authors concluded that cranioplasty within 15 or 30 days after the first craniotomy can reduce the risk of infection and seizure [7]. Most authors are different to this point. Other studies [7, 8] focused on the influence of bone flap storage and the negative influence of parallel shunting [9, 10]. Another study found a lower complication rate of 8% in younger patients (aged 0–39 years) [11]. In the end, because of the retrospective character of most of the studies, the value and influence of different parameters for the operative result are statistically unclear.

In neurosurgery, especially craniofacial surgery, a preoperative CT scan is recommended to diagnose and plan the operative steps to remove the scull. Postoperative, another CT scan is necessary within 48 h to control the operative result. Later, another 3D CT scan is potentially necessary for planning the implant, and a second post-CT scan after implant placement. Computed tomography (CT) is considered the current gold standard. However, a CT always means exposure to radiation for the patient. In young children, this can be a high risk [12]. Numerous studies have investigated the relationship between the frequency of CTs and cancer risk. A clear association can be seen, especially in young children, but it decreases with age [13–17]. In the end, it is sensible to avoid additional unnecessary CT scans. But the basis for the reconstruction of the cranial bone represents 3D imaging, which is an important tool for diagnosis, planning and evaluation of the surgical procedure [12].

### Basics of 3D scanning technology

In addition to CT, alternative imaging techniques that have become increasingly important in recent years. For example, using 3D scanners and photogrammetry systems can digitize the shape of the skull [18–22]. In particular, 3D scanners are increasingly used in everyday clinical practice to create patient-specific orthoses [23, 24].

The scanning of anatomical structures aim to create a 3D digital model that can be used for replacement, implant creation or measurement. The 3D model is a valuable asset for medical staff who require high accuracy.

Today’s most common scanner types are laser scanners and scanners that work with structured light. Structural light scanners emit the light in a defined pattern on the object. Based on this pattern, deformations and distortions of anatomical structures can be detected from various positions. Compared with other imaging methods, this method has a number of significant advantages, including simplicity of use and a low acquisition cost. The main advantage of these products is their ease of handling. In the medical sector, this approach also facilitates the digitization of anatomical structures in patients with limited mobility.

### Scanning and reconstruction

At this point, a distinction must be made between patient-specific and non-specific implants. Specific implants are manufactured analogously to the 3D data for the respective patient. In contrast, non-specific implants, for example, preformed titanium meshes, are adapted to the patient intraoperatively [25, 26] or with the base of average data of the patients. One study [27] suggests that there is a tendency for fewer complications to occur with the use of patient-specific implants. In this retrospective study, 25% (27 out of 108 operations) of complications occurred with titanium mesh, whereas 12.5% (3 out of 24 operations) of complications occurred with a patient-specific implant.

**Fig. 1:**
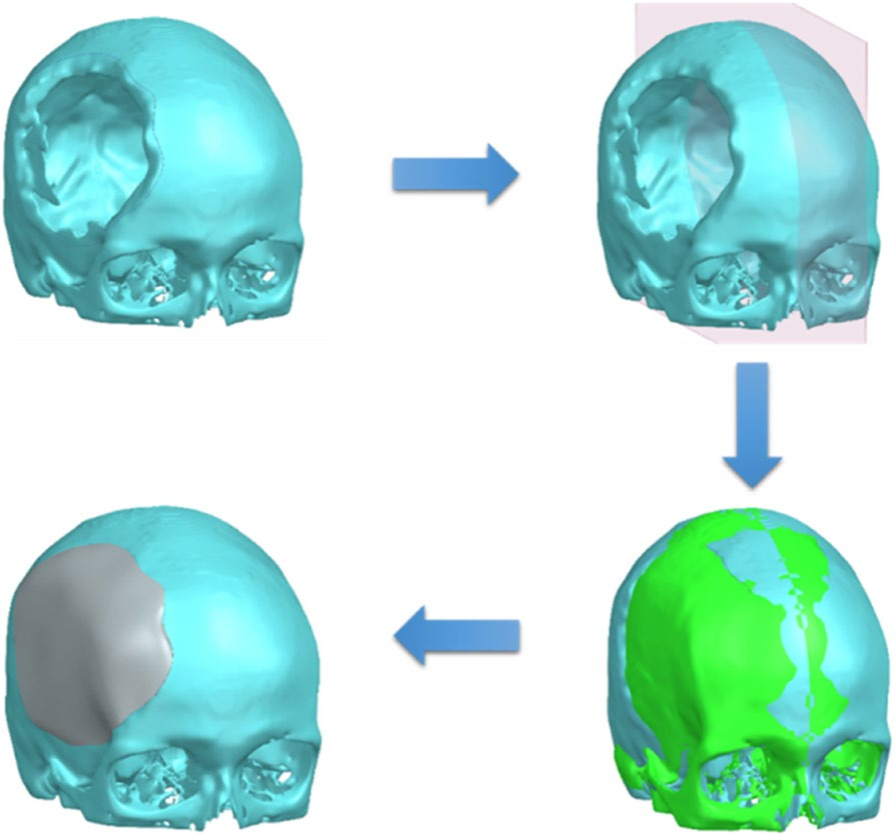
Reflection of the healthy half of the skull as a possible source of inaccuracies

One way to reconstruct the damaged area of the skull is to use the near axial symmetry of the human skull and mirror the intact area of the skull onto the defect (Fig. 1).

This method is based on the simplification of the skull geometry and does not completely represent the anatomical asymmetry of the human skull [28–30]. As a result, the implant does not accurately reconstruct the original skull shape, which can negatively affect the fit with the galea, periosteum and other anatomical structures of the skull. In this case, the reconstructions are based on imaging techniques, such as computed tomography, which are used to create a volume model. Using commercial CAD software, a model of the implant adapted to the shape of the skull is then created by extrapolating the curvature of the skull and transferred to production. However, the conventional reconstruction functions used in this process represent a limitation of the method. For example, the missing contour of the bone flap is manually recreated in a sectional view.

This work aims to develop a new process chain to create patient-specific cranial implants using 3D scanning technology. The goal is to shorten the waiting time for patients needing a cranioplasty and to create a completely individual implant.

## Materials and methods

**Fig. 2:**
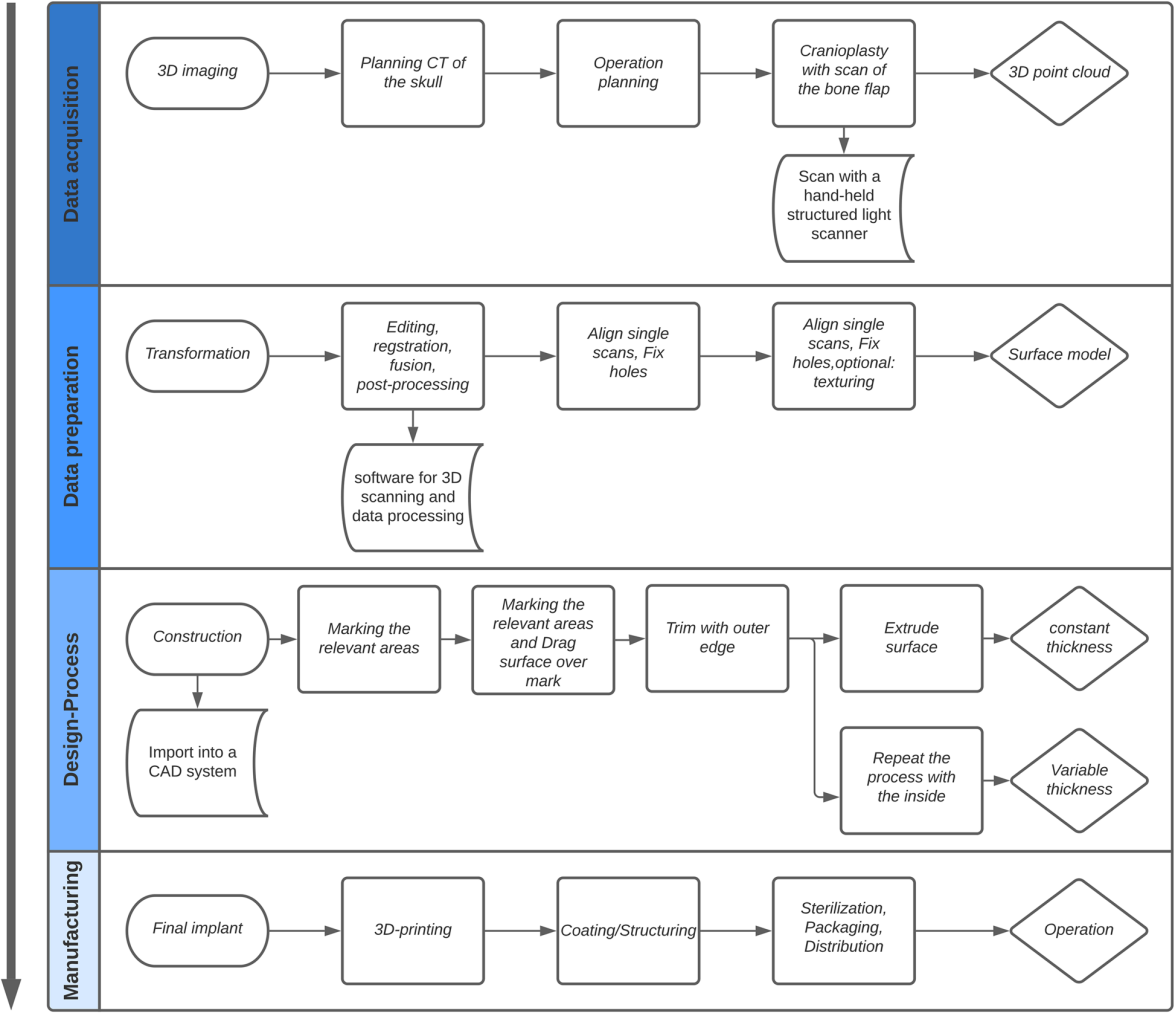
General overview of the new process flow

This part explains the procedure to create an implant. Figure 2 provides a general overview of the individual processes. The real process flow is divided into four subsections, with only data acquisition and implant insertion taking place in the operating room.

### 3D-Scan

In order to be able to exactly reproduce the removed skull contour, the following method is based on an intraoperatively created 3D scan. This scan can easily be performed in the surgical environment. For this purpose, the engineer, in the care of the medical staff, can perform the scan on an operating table.

**Fig. 3:**
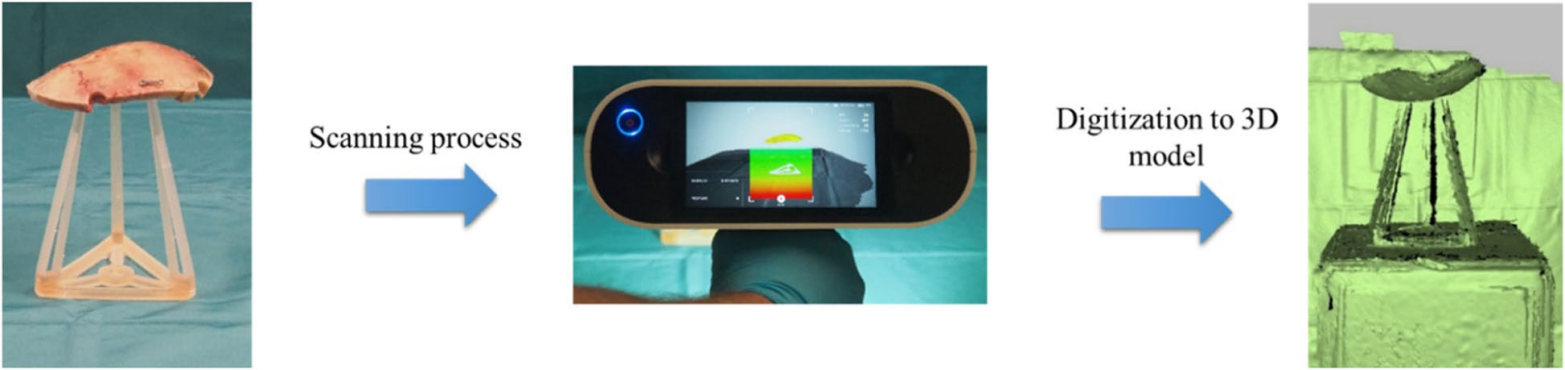
Digitization of a human bone flap

The direct digitization of the extracted bone targets the advantage that the approximate exact skull curvature is now available as information. The method starts with the surgeon’s surgical removal of the bone flap, at which point the bone flap is placed on a sterile surface. As in Fig. 3 the ArtecLeo handheld 3D scanner from the company Arted3D is used for scanning. The accuracy of the outer edge is also the most decisive for the subsequent application because this is where the accuracy of fit is created. For this purpose, a transparent scanning aid is used to be able to record the top and bottom sides at the same time. Depending on the surgical environment and the size of the bone flap, the complete scan takes between 4 and 6 min. The distance to the object is 0.6 m with a texture brightness of 50%.

#### Preparation of the scan data

With the assistance of Artec Studio 15 Professional software, the punctured scan data is converted into a watertight surface model. A monochrome and partially holey object becomes visible, which is composed of a large number of individual frames.

After these processing steps, the result is a watertight surface model.

#### Design process

**Fig. 4:**
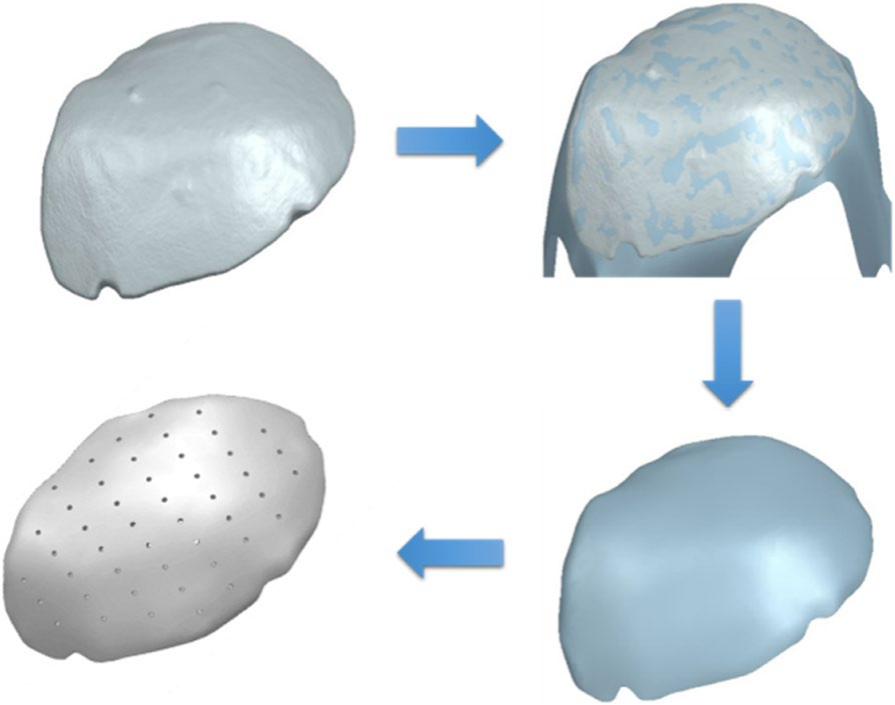
Conversion of the surface scan into a solid model as an implant

The surface body is imported as a mesh body into SiemensNX (SiemensNX, 2019) or another good CAD software. In the first step, all relevant areas are marked, after which an auxiliary surface is stretched over the outside of the bone flap (Fig. 4). The new surface now lies directly on the mesh body and encloses it, exactly replicating the bone flap’s surface curvature. The outer edge of the mesh body is abstracted to the new surface as the trim edge. This creates the first surface of the implant. The two procedures are now available for finishing (Fig. 2). The created surface can be reinforced inwards against the curvature of the skull. This implant then has a constant wall thickness. If a variable thickness is required, analogous to the human bone, the previously described procedure can also be used for the inner surface. Next a surface is fitted on the inner side. This is then trimmed with the outer edge. A connecting surface is created between the two outer surfaces. The final implant is created by joining the three individual surfaces. This procedure can be performed for any bone flap shape. The advantage is the exact reproduction of the curvature of the skull. The result is a solid body which can be further processed with regard to required perforations (drill holes, lattice structures, structural, optimization).

#### Accuracy study

An initial comparison with the associated CT model of the bone flap shows insights into the accuracy of the 3D scan. The GOMInspect software is used for the comparison, whereby at least 50 deviation flags are formed, in addition to a general area comparison.

The area comparison is made on the scan model (actual model), whereby negative deviation indicates shrinkage and positive deviation indicates an increase in materials. A total of 9 bone flaps were examined for the accuracy study of the 3D scanner. At the same time, the usability of the scanner in the surgical environment was to be assessed. The scanned bone flaps came from patients who would no longer have had them inserted. After the scanning process, the bone flaps were CT scanned to compare the two virtual models.

## Results

### 3D-Scan

Compared to other handheld 3D scanners, the ArtecLeo features automatic 3D processing in combination with an integrated touch screen. These features make it possible to follow the scan in real time and correct imperfections. One area for improvement would be the ability to scan the object completely and without gaps. Since inaccuracies in the scan can still occur due to time pressure, changing circumstances and individual handling. However, using the applications for contour closure can assist with these inaccuracies after the initial scan [31].

Another advantage is the easy and wireless handling, which is essential in the operational environment. The initial difficulty was to simultaneously scan the top and bottom of the bone flap and subsequently combine them in the ArtecStudio software. This difficulty represents a potential source of error, as rotating the object can lead to a scan abort. For this purpose, a transparent scanning aid was developed, which is scalable in size and sterilizable. This aid simplifies the subsequent reconstruction of the scan data.

The scanning process only takes a few minutes, although it has become clear that a longer scan does not necessarily lead to better results. It has proven helpful to provide the scanner with distinctive anchor points below the scanning device in order to rearrange the scan in case of a possible scan abort.

### Preparation of the scan data

**Fig. 5:**
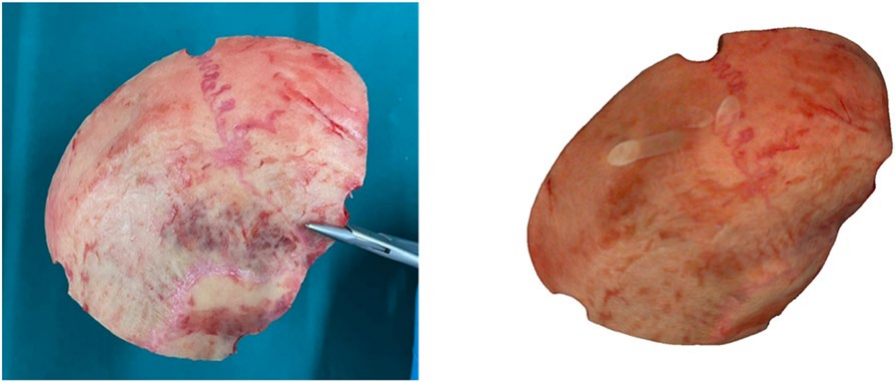
Direct comparison of the real bone (left) flap and the virtual model (right)

After performing the individual steps in ArtecStudio, a watertight surface model is available, which can be seen in Fig. 5. It has been shown that the hand-guided scanner reaches its limits here, particularly in the edge region. The edges appear rounded after preparation and do not reflect the exact fracture edge.

### Design process

The 3D model created here is a completely closed solid, which is ideal for further processing. In some case, the handling of scanned models is still problematic. Which is why the specialist area of reverse engineering is becoming increasingly important. Therefore, this methodology represents an alternative and can be used universally for other bone flap shapes or anatomical structures. In addition, CAD programs always have a range of the same functions, which is why this approach is also possible with other programs.

The conversion of a surface body into a manufactural implant was successful with the help of this technique for all bone flaps examined.

### Accuracy study

These measurement results (Table 1) show a general shrinkage of the scan model compared to the CT model.

**Table 1:** Data from the accuracy study. Overview of the average, maximum and minimum deviation

Patient No	K1	K2	K3	K4	K5	K6	K7	K8	K9
avg. dev [mm]	-1,05	-0,94	-0,81	-1,39	0,53	-0,47	-0,85	-0,30	1,37
max. pos. dev [mm]	-0,39	0,75	0,32	0,55	1,62	0,27	1,07	0,58	3,92
max. neg. dev [mm]	-2,21	-2,56	-2,27	-3,52	-1,06	-1,78	-1,98	-1,13	-1,47

It is noticeable that seven out of nine bone flaps are smaller on average. This phenomenon can be seen in particular on the outer edges of the bone flaps.

**Fig. 6:**
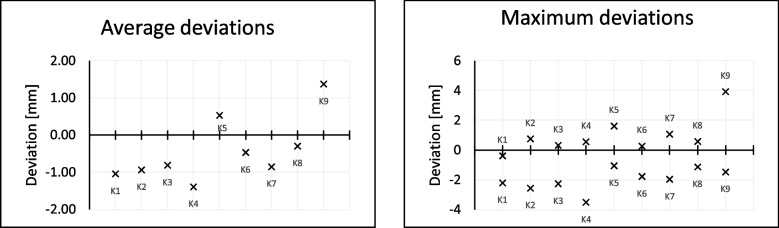
Average (left) and maximum (right) deviations of the bone flaps

**Fig. 7:**
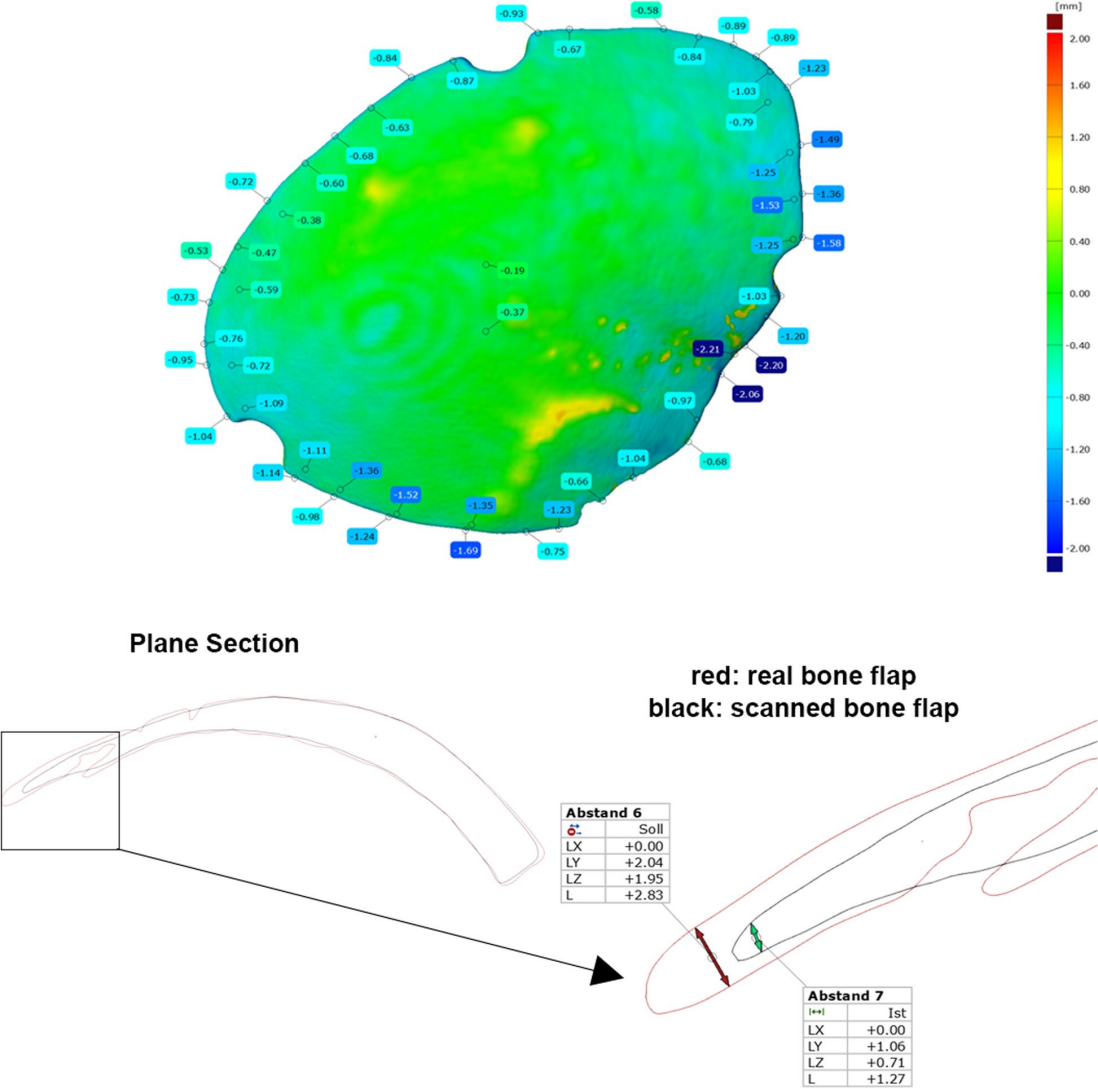
Surface comparison of the real and scanned bone flap (left). Sectional view with recognisable deviation in thin structures (right)

The deviation is particularly evident in thin cross-sections of the bone flaps, as illustrated by the section view (Figs. 6 and 7). For example, the bone flap of test series K8 shows an average deviation of -0.30 and a maximum positive deviation of 0.58. The minimum deviation of -1.13 can be attributed to the difficulty in scanning thin structures. Furthermore, the CT model is created by segmenting the DICOM data.

Here, the bone structure is captured via a targeted gray scale range. Cancellous bone can create holes within the segmented model, which leads to superfluous deviations. Comparing the values of the minimum and maximum deviation, the accuracy of fit seems to be in a moderate range. Compared to the values from the retrospective study by *Moellmann* [32] which examined a total of 42 cranioplasties, their results show that there are implants that achieve significantly higher deviations. Among the cranioplasties studied, twelve implants stand out that have achieved a greater maximum deviation. In addition, there are three implants that achieve a greater minimum deviation. Accordingly, the deviations are not unusual, but this does not mean that they are not debatable and that it is urgent to consider approaches to minimise the deviations. The comparison clearly shows that creating an implant based on a 3D scan is possible.

It should be emphasised that a hand-held structured light scanner, the ArtecLeo, was used here, which is heavily dependent on user guidance. In terms of accuracy, stationary scanners promise significantly more precise results and can be optimally integrated into the surgical environment due to their compact design.

### Manufacturing

**Fig. 8:**
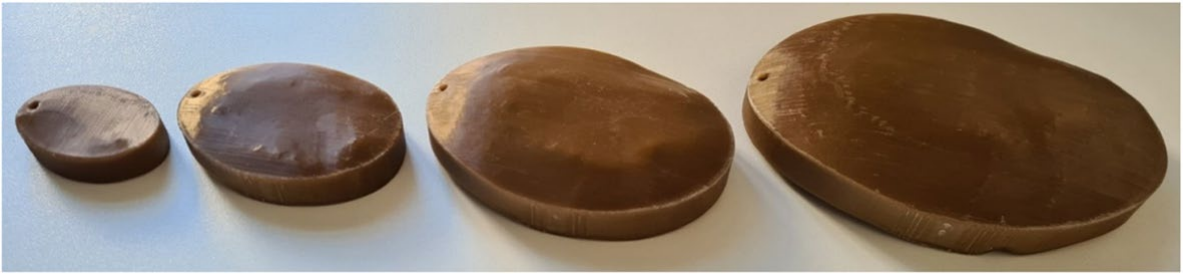
PEEK test specimen

**Fig. 9:**
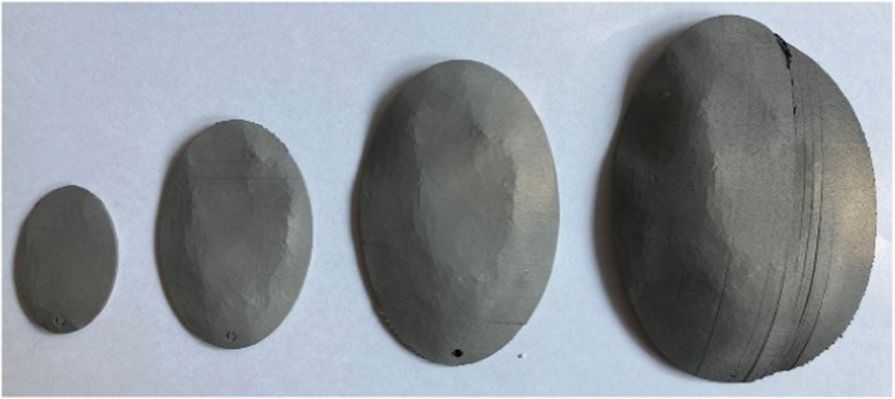
Titanium test specimen

In close consultation with the surgeons, titanium and PEEK were chosen as the materials for the manufacturing process. For this purpose, test specimens are first produced to validate the printing process. The possible printing processes were defined at the beginning for the production of the test specimens and the implants. Titanium is produced using the SLM process (selective laser melting), and PEEK (polyetheretherketone) using the FDM process (fused deposition modelling). With regard to the suitability of the materials for the production of cranial implants, it was necessary to evaluate the biocompatibility and steam sterilisability. PEEK and titanium are entirely suitable for both (Figs. 8 and 9).

After printing the test specimens, the suitability of various measuring methods for assessing the print quality was investigated. Haptic measuring methods proved to be unsuitable, as the measuring probe has difficulty following the free-form geometry, the recording is very time-consuming and the recording of the complete geometry is hardly possible. A 3D scanner with high resolution proved to be suitable for recording. In addition, a µ-CT was used for the recording, especially of the PEEK printed parts.

**Fig. 10:**
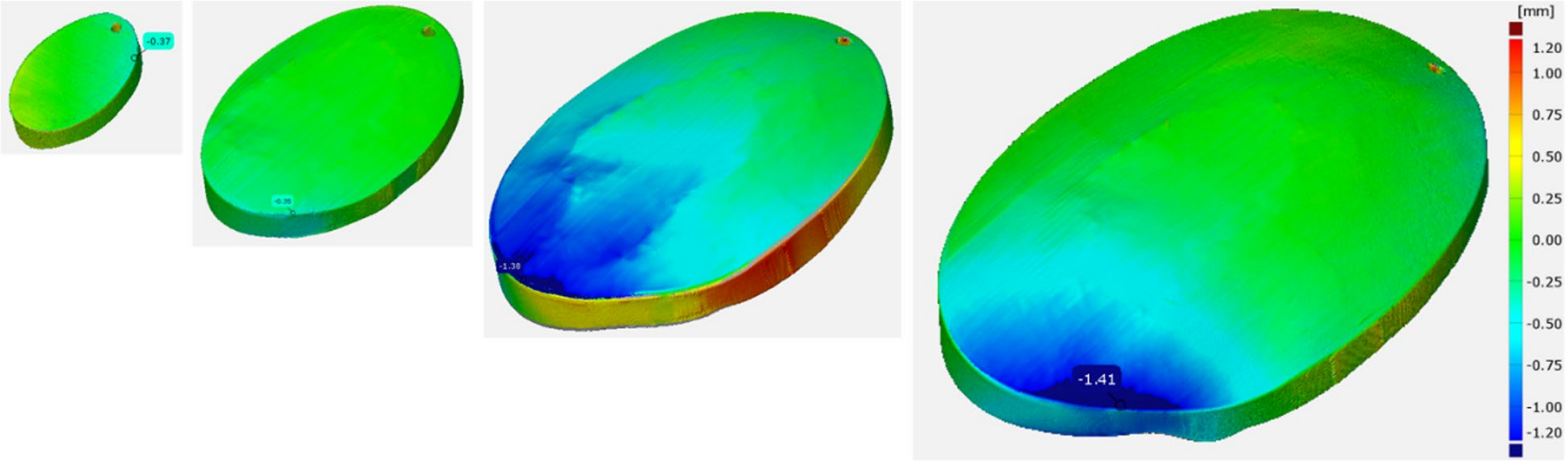
Measurement of PEEK test specimen

Figure 10 shows the results of the nominal/actual comparison of the PEEK test specimens. It can be seen that the deviations increase with increasing component size (maximum at 1.41 mm). Green areas indicate values close to the nominal geometry, and blue and red areas indicate large deviations in the negative and positive. The influence of the annealing process on the deviations was also analysed during the investigations. However, this is less than the influence of the printing process itself.

**Fig. 11:**
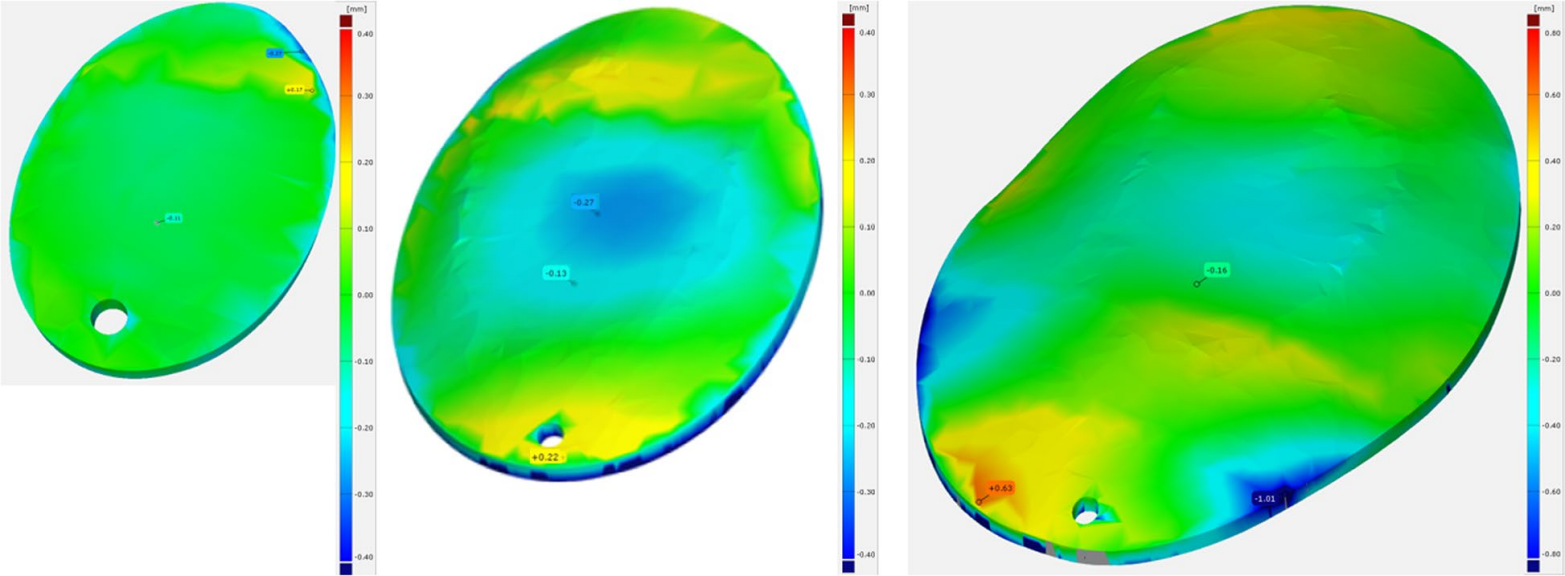
Measurement of Titanium test specimen

Figure 11 Measurement of Titanium test specimen shows the results of the nominal/actual comparison of the titanium test specimens. The deviations also increase with increasing component size. However, the maximum is only 0.63 mm and thus less than half of the PEEK samples.

**Fig. 12:**
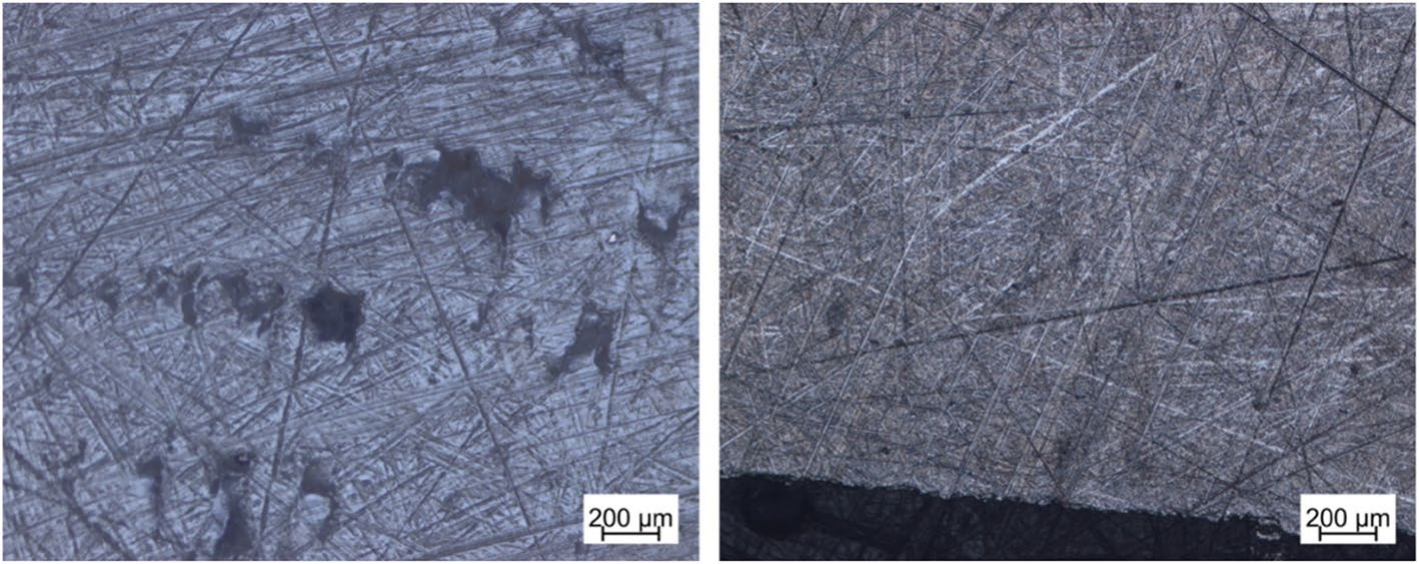
Micrographs left: PEEK, right: titanium

In order to assess the component quality, independent of the dimensional accuracy, micrographs were made for the pressure parts made of PEEK and titanium. For this purpose, parts of the samples were embedded in plastic and then ground on grinding wheels. Figure 12 shows the microscopic image (both 50 × magnification) of the microsection of PEEK on the left and the microsection of titanium on the right. In the case of PEEK, clear holes, also known as blowholes, can be seen. No holes are visible in the micrograph of titanium, and, according to the manufacturers of the SLM machines, none are present.

**Fig. 13:**
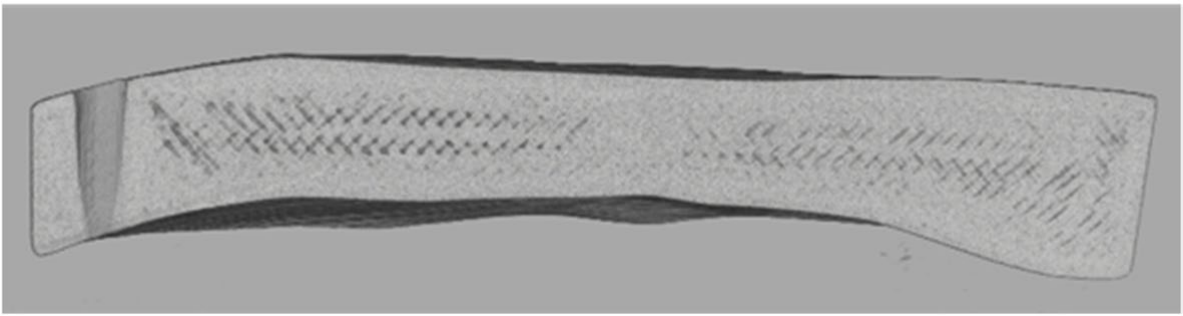
CT image for the selection of internal defects (PEEK)

As an additional method of assessing component quality, CT images were taken to select internal defects. Figure 13 shows an example of a CT image of a PEEK implant. This confirms the previously selected internal defects in PEEK. The defects show clear preferential directions, which are due to the production in the FDM process.

The crack penetration and fluorescence methods did not provide reliable results; the layered white structure shows cracks that are not cracks (cracked?).

The implants made of titanium do not have any internal defects, so the pressure alignment is irrelevant with regard to directional dependencies and cavities. On the other hand, the implants made of PEEK, show clear defects (especially in the center of the component). Short pressure paths, partial melting of the previous layer and suitable travel paths of the extruder inside the component can minimise the number of defects.

## Discussion

Overall, this methodology represents an alternative option for the design of patient-specific skull implants based on an asymmetric reflection. The design sequence in the CAD software is fixed and can be quickly used for the individual bone flap by minor adjustments (selection of surfaces for the individual features).

Performing the 3D scan in the intraoperative environment requires some prior knowledge with the scanning system. However, once a certain basic experience prevails, the digitization of the bone flap can be performed well. The subsequent processing with ArtecStudio also requires some prior knowledge, but usable results are very likely if the individual process steps are followed. At this point, it should be investigated what influence various setting parameters of the software have with regard to detail accuracy, in order to obtain the real bone flap as a model as far as possible.

The subsequent design can also be carried out with other CAD programs, although the functions may differ. The individual steps used here are convincing in their ease of use and regarding the reliability of obtaining a suitable result. A simple conversion of the surface body into a solid body with simple reverse engineering functions proved to be unsuitable because the bone flap deformed too much. Therefore, the methodology described here is a safe way to create an implant.

The documented deviations can be attributed to the scanner’s resolution in combination with a thin outer edge of the bone flap. They can, however, be corrected with a better scanning system.

A stationary scanning system with a higher resolution promises to capture an ideal 3D model and works reliably without disrupting the surgical procedure.

Since these are complex free-form surfaces that ideally map the curvature of the skull, the use of additive manufacturing techniques will follow. The first prototypes have already been produced with PEEK and titanium. Further research and clinical studies are needed to explore the material properties and possible modifications of cranioplastic applications.

## Conclusion

In summary, it is possible to create an accurate image of the bone flap with a hand-held 3D scanner during surgery. With knowledge of 3D post-processing and design, an implant can be created. The accuracy study has shown that the corresponding implant has some deviations but is well within the acceptable range compared to literature values. With a stationary scanning system, with a more accurate resolution, these inaccuracies can be eliminated. Ultimately, this approach promises a faster restoration of the patient, depending on the scanning system also a more accurate image of the bone flap and the elimination of a CT and the associated radiation exposure.

## Data Availability

The data are given in the paper and can be received on request.
